# Primary Neuroendocrine Tumor of the Liver With Papillary Features in a Multivisceral Transplant Patient

**DOI:** 10.7759/cureus.17394

**Published:** 2021-08-23

**Authors:** Jaffar Khan, Lin Jingmei

**Affiliations:** 1 Pathology and Laboratory Medicine, Indiana University School of Medicine, Indianapolis, USA

**Keywords:** ciliated papillary features, primary liver neuroendocrine tumor, multivisceral transplant, poorly differentiated neuroendocrine carcinoma, hepatic neuroendocrine

## Abstract

Primary neuroendocrine tumors (NETs) of the liver are rare and difficult to distinguish from other liver tumors such as cholangiocarcinoma and hepatocellular carcinoma. The patient was initially diagnosed with a NET of the liver in 2007. However, the origin of the cancer was not clear, that is, whether it was primary or originated from the gastrointestinal tract. Although the patient underwent partial hepatectomy, he suffered hepatic artery injury, resulting in biliary strictures. The patient eventually became untreatable and developed cirrhosis, a frozen abdomen. He received multivisceral transplantation in May 2019 and received the liver, duodenal-pancreatic complex, spleen, small bowel, and right colon. After the transplantation, the patient did well overall. More recently, he presented with food poisoning and underwent evaluation, and was found to have a mass in the liver. The liver mass was biopsied and revealed a poorly differentiated primary NET (grade 2) with ciliated papillary structures.

## Introduction

Hepatic neuroendocrine tumors (NETs) are a rare type of tumor arising from the neuroendocrine system. Most of the NETs in the liver are metastatic and primarily originate in the gastrointestinal tract, followed by the lung [1]. Primary hepatic NETs are extremely rare, first described by Edmondson in 1958, with only less than 100 cases reported in the literature [2,3]. It is tough to diagnose NETs without biopsy based on the imaging and clinical picture [4]. The incidence rate of NETs in the United States is 6.25 cases per 100,000 individuals per year, according to the current literature [5]. Primary NETs do not display any gender predominance [6].

## Case presentation

**Figure 1:**
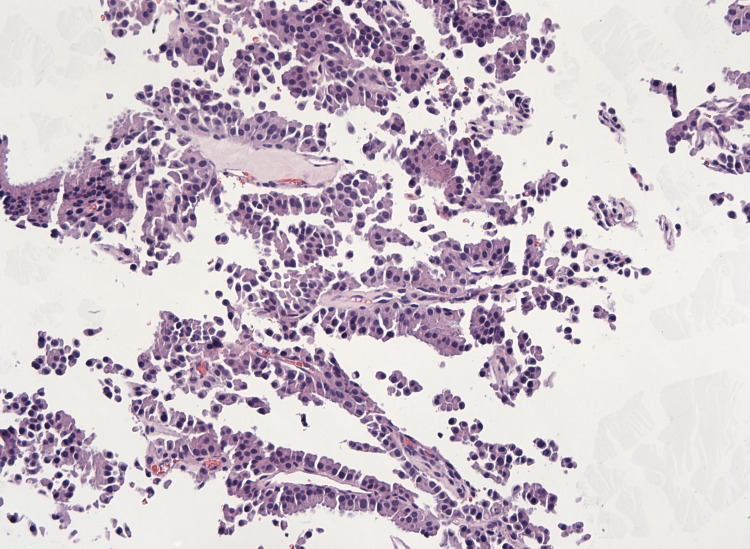
Hematoxylin and eosin (10×) staining showing neuroendocrine tumor with papillary features.

**Figure 2:**
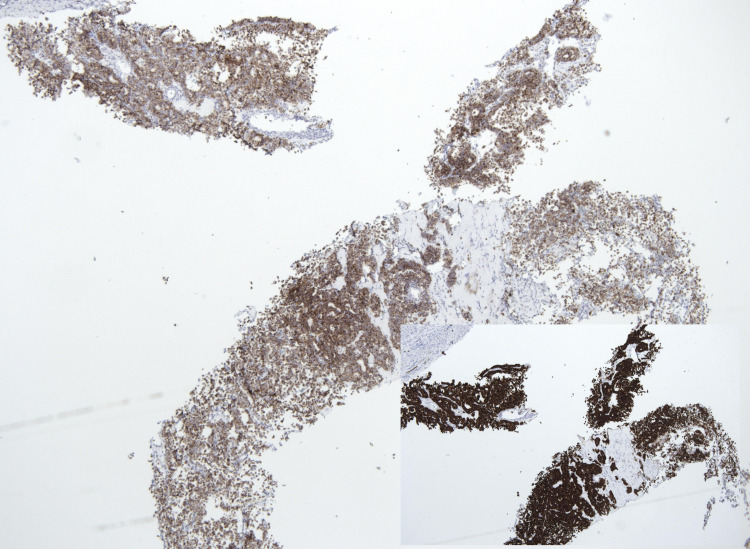
Immunohistochemical stains: chromogranin. The insert shows synaptophysin staining (2×).

A 65-year-old male with a history of multivisceral transplant presented with food poisoning and was found to have a liver mass on imaging. The patient was initially diagnosed with a NET of the liver in 2007. The origin of the tumor was not clear, that is, whether it was primary or originated from the gastrointestinal tract. Although the patient underwent partial hepatectomy, he experienced hepatic artery injury which caused biliary strictures and eventually became untreatable; this led to cirrhosis and a frozen abdomen, requiring multivisceral transplantation. He received multivisceral transplantation in May 2019 which included the liver, duodenal-pancreatic complex, spleen, small bowel, and right colon. After the surgery, he did well overall. Recently, he presented with food poisoning, and on evaluation, was found to have a mass in the liver. The liver mass was biopsied which showed a neoplastic process with relatively uniform cellular features. The neoplastic cells were arranged in a papillary configuration. No atypical mitosis was identified (Figure 1). Immunohistochemical stains were obtained with appropriate working controls to subclassify the tumor. The neoplastic cells were strongly and diffusely positive for synaptophysin and chromogranin (Figure 2), and focally positive for cytokeratin 7 and 20. The cells were negative for thyroid transcription factor 1 (TTF1), GATA binding protein 3 (GATA3), paired-box gene 8 (PAX8), and caudal-type homeobox 2 (CDX2). The Ki67 proliferation index was 10%. Thus, the diagnosis of an intermediate-grade NET (G2) was rendered. The patient had no other obvious sites of disease and was asymptomatic. Additionally, he had no history of weight loss, fever, jaundice, or bleeding.

## Discussion

The World Health Organization uses the number of mitosis per 10 high-power fields to grade gastrointestinal NETs or the percentage of neoplastic cells immunolabeled by the proliferation marker Ki67. These markers indicate the rate of neoplastic proliferation and can predict the clinical outcome. Gastro-entero-pancreatic tumors are classified into three types: well-differentiated tumors of low-grade malignancy (grade 1), well-differentiated tumors of intermediate-grade neoplasms (grade 2), and poorly differentiated neoplasms (grade 3) [7]. These tumors are rare, and it is difficult to differentiate them from other common hepatic malignancies [8].

Only 6.8% of hepatic NETs present with typical carcinoid syndrome symptoms such as skin flushing, abdominal pain, and diarrhea. These tumors are mostly diagnosed because of their mass effect, not their carcinoid symptoms. Primary hepatic NETs grow very slowly and are diagnosed at a very advanced stage as a mass [9]. Most cases of hepatic NETs are misdiagnosed as hepatocellular carcinomas on imaging [10]. Both primary and metastatic NETs have similar findings on radiological imaging [11]. Therefore an extensive search for extrahepatic NETs should be done using computed tomography, magnetic resonance imaging, positron emission tomography, colonoscopy, and bronchoscopy, including other studies to exclude extrahepatic origins [12].

Primary NETs can appear gray to yellow in color grossly and have well-demarcated masses with multiple irregular hemorrhagic lesions with or without cystic areas [13]. These tumors show nested, trabecular, insular, or mixed patterns of cell growth on histomorphology. In our case, the liver biopsy showed a neoplastic process with relatively uniform cellular features. The neoplastic cells were arranged in a papillary configuration. No atypical mitosis was identified. Immunohistochemical stains were performed with appropriate working controls to subclassify the tumor. The neoplastic cells were strongly and diffusely positive for synaptophysin and chromogranin, and focally positive for cytokeratin 7 and 20. The cells were negative for TTF1, GATA3, PAX8, and CDX2. The Ki67 proliferation index was 10%. Immunohistochemical staining ruled out the possibility of the tumor originating from any other site other than the liver. The staining pattern best fits with the diagnosis of NETs. It was challenging to diagnose the case with a papillary pattern without obtaining immunohistochemical stains.

Although there are no treatment guidelines for primary hepatic NETs, surgical resection is the treatment of choice, including wedge resection or lobectomy [14]. The risk of recurrence of primary hepatic carcinoid tumors after resection remains unknown [15]. In patients with unresectable diseases, palliative options such as systemic 5-fluorouracil, hepatic artery embolization, and octreotide therapy are available [16-18].

## Conclusions

Primary hepatic NETs are extremely rare, and there is no reported case of a primary hepatic NET with ciliated papillary features in transplanted liver cases. The diagnosis of primary hepatic NETs is a real challenge, and it is very hard to distinguish these tumors from hepatocellular carcinoma and cholangiocarcinoma. The presence of ciliated papillary features adds more difficulty, making it harder to diagnose such cases. This case highlights the importance of histomorphology, and, therefore, the pathologists need to look for these features and always obtain the needed immunohistochemical stains in proper settings to rule out neuroendocrine carcinoma.

## References

[1] Yang Z, Klimstra DS, Hruban RH, Tang LH (2017). Immunohistochemical characterization of the origins of metastatic well-differentiated neuroendocrine tumors to the liver. Am J Surg Pathol.

[2] Edmondson HA (1958). Tumor of the liver and intrahepatic bile duct. Atlas of Tumor Pathology.

[3] Lin CW, Lai CH, Hsu CC, Hsu CT, Hsieh PM, Hung KC, Chen YS (2009). Primary hepatic carcinoid tumor: a case report and review of the literature. Cases J.

[4] Kumar A, Kalonia T, Bharati V, Gupta A (2020). Primary neuroendocrine tumor of liver: an eye opener for a pathologist. J Family Med Prim Care.

[5] Knox CD, Anderson CD, Lamps LW, Adkins RB, Pinson CW (2003). Long-term survival after resection for primary hepatic carcinoid tumor. Ann Surg Oncol.

[6] Quartey B (2011). Primary hepatic neuroendocrine tumor: what do we know now?. World J Oncol.

[7] Klimstra DS, Modlin IR, Coppola D, Lloyd RV, Suster S (2010). The pathologic classification of neuroendocrine tumors: a review of nomenclature, grading, and staging systems. Pancreas.

[8] Xia Y, Zhang L, Wu H, Qiao L, Xia L (2020). Primary hepatic neuroendocrine tumor with multiple liver metastases: a case report with literature review. J Int Med Res.

[9] Jia C, Zhang Y, Xu J, Sun K (2012). Experience in primary hepatic neuroendocrine tumor. Turk J Gastroenterol.

[10] Kellock T, Tuong B, Harris AC, Yoshida E (2014). Diagnostic imaging of primary hepatic neuroendocrine tumors: a case and discussion of the literature. Case Rep Radiol.

[11] Baek SH, Yoon JH, Kim KW (2013). Primary hepatic neuroendocrine tumor: gadoxetic acid (Gd-EOB-DTPA)-enhanced magnetic resonance imaging. Acta Radiol Short Rep.

[12] Landen S, Elens M, Vrancken C, Nuytens F, Meert T, Delugeau V (2014). Giant hepatic carcinoid: a rare tumor with a favorable prognosis. Case Rep Surg.

[13] Pilichowska M, Kimura N, Ouchi A, Lin H, Mizuno Y, Nagura H (1999). Primary hepatic carcinoid and neuroendocrine carcinoma: clinicopathological and immunohistochemical study of five cases. Pathol Int.

[14] Yalav O, Ülkü A, Akçam TA, Demiryürek H, Doran F (2012). Primary hepatic neuroendocrine tumor: five cases with different preoperative diagnoses. Turk J Gastroenterol.

[15] Schwartz G, Colanta A, Gaetz H, Olichney J, Attiyeh F (2008). Primary carcinoid tumors of the liver. World J Surg Oncol.

[16] Andreola S, Lombardi L, Audisio RA (1990). A clinicopathologic study of primary hepatic carcinoid tumors. Cancer.

[17] Krishnamurthy SC, Dutta V, Pai SA (1996). Primary carcinoid tumor of the liver: report of four resected cases including one with gastrin production. J Surg Oncol.

[18] Wängberg B, Nilsson O, Johanson V V (1997). Somatostatin receptors in the diagnosis and therapy of neuroendocrine tumor. Oncologist.

